# Supplementary material for: Introduction of Solid Foods in Preterm Infants and Its Impact on Growth in the First Year of Life—A Prospective Observational Study
Source: Nutrients. 2024 Jun 28;16(13):2077. doi: 10.3390/nu16132077 (PMC11242969; doi:10.3390/nu16132077)
Supplement: Supplementary file 1 [file nutrients-16-02077-s001.zip › nutrients-3074974-supplementary.pdf]

Figure S1. Study flow.

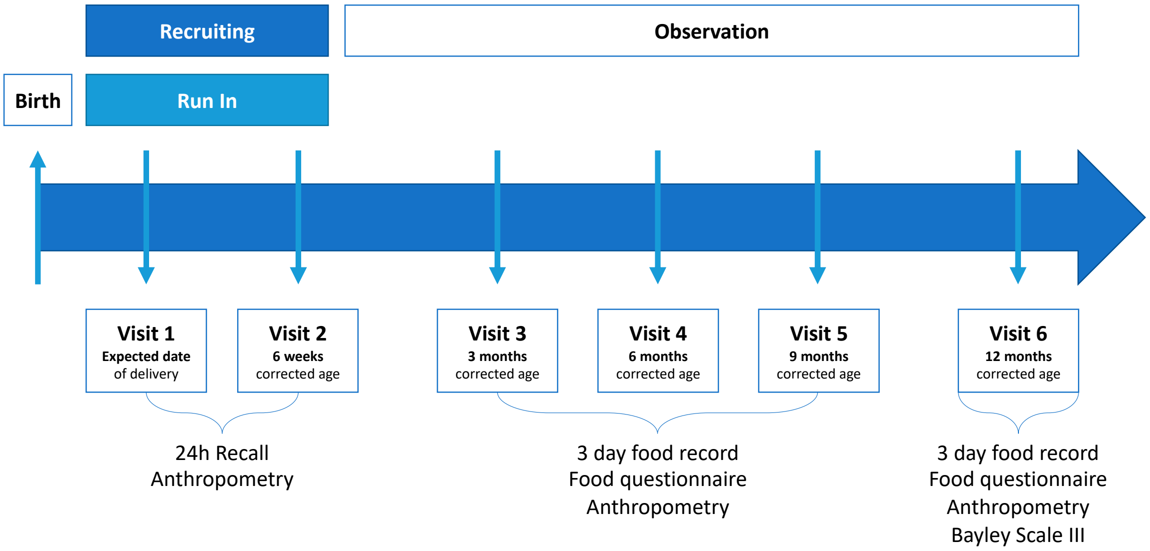

Table S1. Anthropometric measurements

Parameter	Early group (n=115)	Late group (n=82)	p-value
Weight, g			
Birth	926 (±254)	881 (±262)	0.214
Estimated date of birth	3223 (±530)	3322 (±457)	0.237
6 weeks corrected age	4486 (±703)	4339 (±678)	0.181
3 months corrected age	5641 (±858)	5448 (±847)	0.130
6 months corrected age	7203 (±1083)	6870 (±1149)	0.065
12 months corrected age	9253 (±1423)	8851 (±1428)	0.098
Weight z-score			
Birth	0.01 (±0.83)	−0.09 (±0.94)	0.469
Estimated date of birth	−0.85 (±1.04)	−0.94 (±0.89)	0.325
6 weeks corrected age	−0.64 (±1.15)	−0.96 (±1.08)	**0.043**
3 months corrected age	−0.91 (±1.23)	−1.07 (±1.22)	0.291
6 months corrected age	−0.73 (±1.29)	−0.98 (±1.43)	0.310
12 months corrected age	−0.31 (±1.29)	−0.59 (±1.35)	0.149
Length, cm			
Birth	34.8 (±3.2)	33.9 (±3.5)	0.074
Estimated date of birth	49.2 (±2.7)	49.7 (±2.4)	0.205
6 weeks corrected age	54.8 (±2.6)	54.0 (±2.9)	0.095
3 months corrected age	59.8 (±2.8)	59.1 (±2.9)	**0.041**
6 months corrected age	66.9 (±2.9)	65.8 (±2.9)	**0.008**
12 months corrected age	75.0 (±3.1)	74.1 (±3.3)	0.053
Length z-score			
Birth	0.12 (±0.98)	−0.13 (±0.98)	0.136
Estimated date of birth	−0.91 (±1.13)	−1.05 (±1.14)	0.481
6 weeks corrected age	−0.60 (±1.34)	−1.02 (±1.39)	**0.036**
3 months corrected age	−0.59 (±1.33)	−0.82 (±1.35)	0.131
6 months corrected age	−0.10 (±1.29)	−0.39 (±1.22)	0.089
12 months corrected age	−0.27 (±1.18)	−0.44 (±1.22)	0.274
Head circumference			
Birth	24.6 (±2.2)	24.2 (±2.5)	0.165
Estimated date of birth	34.4 (±1.6)	34.8 (±1.5)	0.146
6 weeks corrected age	37.4 (±1.5)	37.2 (±1.7)	0.532
3 months corrected age	39.7 (±1.5)	39.5 (±1.9)	0.299
6 months corrected age	42.6 (±1.8)	42.4 (±1.7)	0.208
12 months corrected age	45.5 (±1.8)	45.0 (±2.1)	0.066
Head circumference z-score			
Birth	0.27 (±0.88)	0.08 (±0.95)	0.199
Estimated date of birth	−0.48 (±1.09)	−0.53 (±1.15)	0.667
6 weeks corrected age	−0.38 (±1.30)	−0.56 (±1.39)	0.365
3 months corrected age	−0.44 (±1.25)	−0.49 (±1.46)	0.813
6 months corrected age	−0.31 (±1.40)	−0.26 (±1.29)	0.935
12 months corrected age	−0.18 (±1.44)	−0.38 (±1.47)	0.301
BMI			
Estimated date of birth	13.2 (±1.2)	13.4 (±1.4)	0.463
6 weeks corrected age	14.9 (±1.4)	14.8 (±1.3)	0.492
3 months corrected age	15.7 (±1.6)	15.5 (±1.4)	0.559
6 months corrected age	16.0 (±1.5)	15.8 (±2.0)	0.268
12 months corrected age	16.4 (±1.9)	16.1 (±1.7)	0.293

Continuous data are presented as the mean and standard deviation in parentheses. Differences between study groups were calculated using the Mann-Whitney-U-test. p-vales <0.05 were considered statistically significant.

Figure S2. Dependency plot – Effect size of weeks corrected age at starting solid food on length at 12 months corrected age.

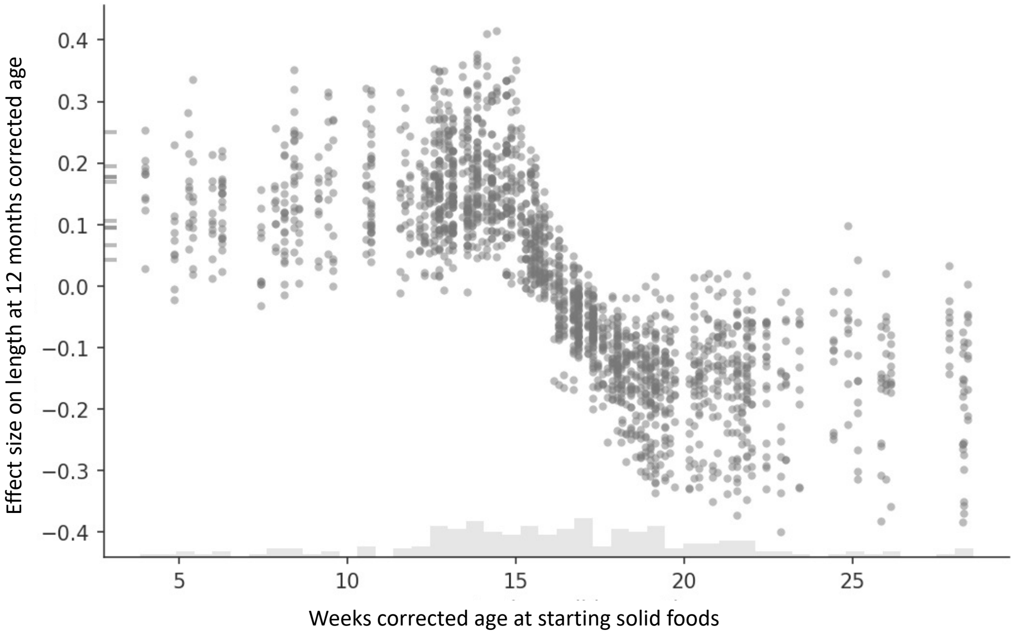

Table S2. Weight, length, and head circumference z-score in infants with comorbidities.

**Parameter**	**Infants without NEC, BPD, IVH** (n=132)	**NEC ≥ grade II** (n = 11)	**BPD** (n = 37)	**IVH ≥ grade II** (n = 29)
Weight z-score				
Birth	0.02 (±0.88)	−0.49 (±1.03)	−0.22 (0.92)	−0.09 (±0.89)
Estimated date of birth	−0.78 (±1.00)	−1.30 (±0.83)	−1.17 (0.95)	−1.12 (±1.08)
6 weeks corrected age	−0.57 (±1.13)	−1.80 (±1.28)*	−1.18 (0.86)*	−0.96 (±1.16)
3 months corrected age	−0.75 (±1.24)	−1.74 (±1.15)*	−1.39 (1.06)*	−1.22 (±1.09)
6 months corrected age	−0.61 (±1.30)	−1.58 (±1.19)	−1.23 (1.44)	−1.17 (±1.27)
12 months corrected age	−0.18 (±1.21)	−1.26 (±1.06)*	−0.82 (1.52)*	−0.88 (±1.26)
Length z-score				
Birth	0.06 (±0.97)	−0.43 (±1.41)	−0.13 (±1.04)	0.02 (±1.05)
Estimated date of birth	−0.77 (±1.08)	−1.42 (±0.95)	−1.61 (±1.02)*	−1.29 (±1.45)
6 weeks corrected age	−0.54 (±1.33)	−2.05 (±1.59)*	−1.45 (±1.09)*	−0.85 (±1.54)
3 months corrected age	−0.45 (±1.32)	−1.43 (±1.25)*	−1.32 (±1.13)*	−0.79 (±1.42)
6 months corrected age	0.01 (±1.22)	−0.81 (±1.21)	−0.83 (±1.03)*	−0.42 (±1.43)
12 months corrected age	−0.19 (±1.15)	−0.84 (±1.33)	−0.72 (±1.06)	−0.37 (±1.34)
Head circumference z-score				
Birth	0.26 (±0.87)	−0.35 (±0.92)	0.01 (1.01)	0.13 (±1.03)
Estimated date of birth	−0.16 (±0.93)	−1.41 (±0.71)*	−1.18 (1.01)*	−1.32 (±1.52)*
6 weeks corrected age	−0.05 (±1.17)	−1.69 (±1.16)*	−1.12 (1.03)*	−1.32 (±1.65)*
3 months corrected age	−0.08 (±1.14)	−1.65 (±0.91)*	−0.92 (1.17)*	−1.25 (±1.78)*
6 months corrected age	0.05 (±1.21)	−1.27 (±0.89)*	−0.73 (1.18)*	−1.02 (±1.71)*
12 months corrected age	0.11 (±1.30)	−1.14 (±0.64)*	−0.73 (1.24)*	−1.10 (±1.95)*

Continuous data are presented as the means and standard deviations in parentheses. Z-scores were compared between infants with and without comorbidities using a Mann-Whitney-U test. p-vales <0.05 were considered statistically significant. Significant differences are marked with a *.

BPD – bronchopulmonary dysplasia defined as oxygen demand >36+0, IVH – intraventricular hemorrhage, NEC – necrotizing enterocolitis

Table S3. Machine learning model including influential factors for the prediction of age at starting solid foods.

	**Age at starting solid foods**	
	$R^2 = 0.048$	
	Effect size	*p-value*
BPD	0.31	0.002
Sex	0.27	0.115
Nutrition at 6 weeks corrected age	0.24	0.094
Highest parental education	0.15	0.703
Gestational age	0.14	0.354
Maternal country of birth	0.12	0.422
Maternal age	0.11	0.735
IVH	0.03	0.818
NEC	0.03	0.662
